# Mechanisms of arrhythmia termination during acute myocardial ischemia: Role of ephaptic coupling and complex geometry of border zone

**DOI:** 10.1371/journal.pone.0264570

**Published:** 2022-03-15

**Authors:** Ning Wei, Elena G. Tolkacheva

**Affiliations:** 1 Department of Mathematics, Purdue University, West Lafayette, IN, United States of America; 2 Department of Biomedical Engineering, University of Minnesota, Minneapolis, MN, United States of America; Universiteit Gent, BELGIUM

## Abstract

Myocardial ischemia occurs when blood flow to the heart is reduced, preventing the heart muscle from receiving enough oxygen required for survival. Several anatomical and electrophysiological changes occur at the ischemic core (IC) and border zone (BZ) during myocardial ischemia, for example, gap junctional remodeling, changes in ionic channel kinetics and electrophysiologic changes in cell excitability, which promote the development of cardiac arrhythmia. Ephaptic coupling (EpC), which is an electrical field effect developed in the shared cleft space between adjacent cells, has been suggested to rescue the conduction when gap junctions are impaired, such as myocardial ischemia. In this manuscript, we explored the impact of EpC, electrophysiological and anatomical components of myocardial ischemia on reentry termination during non-ischemic and ischemic condition. Our results indicated that EpC and BZ with complex geometry have opposite effects on the reentry termination. In particular, the presence of homogeneous EpC terminates reentry, whereas BZ with complex geometry alone facilitates reentry by producing wave break-up and alternating conduction block. The reentry is terminated in the presence of homogeneous or heterogeneous EpC despite the presence of complex geometry of the BZ, independent of the location of BZ. The inhibition of reentry can be attributed to a current-to-load mismatch. Our results points to an antiarrhythmic role of EpC and a pro-arrhythmic role of BZ with complex geometry.

## Introduction

Myocardial ischemia is a medical condition resulting from critical coronary artery obstruction, which can lead to serious complications, including but not limiting to heart attack, arrhythmia and heart failure. It is one of the leading causes of sudden cardiac death worldwide.

Altered electrophysiological and anatomical properties that occurred during myocardial ischemia lead to significant changes in the shape of action potential and its propagation [1–3]. It has been demonstrated that the cardiac conduction would be slow and discontinuous due to an alternation in the dynamics of ionic channels and gap junction remodeling [1–3]. For the past decades, hypothesis for an alternative mechanism of electrical propagation in the heart, such as ephaptic coupling (EpC) has been proposed. EpC is an electric field effect developed at the cleft space between cells, which makes propagation possible in the absence of gap junctions [4, 5]. Several experimental and numerical studies show that EpC can help sustain conduction when gap junctional coupling is compromised [6–15]. However, there are several studies that demonstrate a pro-arrhythmic role of EpC. For instance, numerical study [16] shows that EpC (cleft width ranges from 7 nm to 17 nm) gives rise to a phase 2 reentry in patients suffering from Brugada syndrome. Authors of [17] show that attenuation of EpC by reducing extracellular Na^+^ concentration (${\left[\text{Na}\right]}_{\text{o}}^{+}$) or facilitating perinexal expansion delays the onset of arrhythmia during metabolic ischemia, which indicates a proarrhythmic role of EpC. While the direct experimental evidence demonstrating the existence of EpC is still missing, there are several experimental studies on changes of perinexal structure under various conditions [17–21], which possesses the structural requirements for EpC. Studies in [19] show that the mean perinexal width (*W*_*p*_) over all combined perfusate groups is larger during no flow ischemia under elevated [Na^+^]_o_ and extracellular Ca^2+^ concentration. In [20], the authors demonstrate that *W*_*p*_ can modulate the relationship between gap junctions and conduction velocity (CV). In particular, when *W*_*p*_ is large, CV is more sensitive to changes in perfusate composition and connexin 43 (Cx43). When *W*_*p*_ is small, CV is less sensitive to the aftermentioned changes. However, the impact of EpC on arrhythmia termination in ischemic heart has never been investigated.

Several regions are noted during myocardial ischemia: ischemic core (IC), where irreversible death of heart muscle occurs due to prolonged myocardial ischemia; normal zone (NZ), in which all cells are under normal electrophysiological conditions; and border zone (BZ) that lies in between IC and NZ, which is created due to different blood and oxygen levels. Recent experimental findings reveal that BZ has a complex geometry, where IC penetrates into NZ in a “finger-like” fashion [22]. Authors of [15] showed that more frequent, deeper, and wider “fingers” in the BZ promote the conduction from NZ to IC. In contrast, larger size of IC impedes the cardiac conduction. However, the detailed impact of “finger-like” geometry of BZ [22] on arrhythmia termination is still unclear.

In this manuscript, we aim to explore the impact of EpC on arrythmogenesis during non-ischemic and ischemic condition. Specifically, we aim to investigate the contribution of EpC, electrophysiological and anatomical components of myocardial ischemia on reentry termination. See Table 1 for overall organization and main results of our manuscript.

**Table 1:** Arrhythmia termination during acute myocardial ischemia: Role of EpC and complex geometry of BZ.

Myocardial ischemia			Gap junctions	EpC	Figure	Arrhythmia termination	
IC location	BZ geometry	Conditions				Initial condition	Termination
**Effect of EpC on arrhythmia**							
No ischemia			100%	No EpC	Fig 2		Arrhythmia 1
				Homogeneous (*d*_cleft_ = 8 nm)	Fig 3	Arrhythmia 1	Yes
				Homogeneous (*d*_cleft_ = 13 nm)	S1 Fig		Yes (longer time)
				Homogeneous (*d*_cleft_ = 23 nm)	S2 Fig		
			1%	No EpC	Fig 4		Arrhythmia 2
				Homogeneous (*d*_cleft_ = 8 nm)	Fig 5	Arrhythmia 2	Yes
**Effect of myocardial ischemia on arrhythmia**							
No IC/BZ		Hyperkalemia	100%	No EpC	Fig 6	Arrhythmia 1	Yes
		Acidosis/Anoxia			No		No
		Uncoupling	0.5%		Fig 7		No (multiple reentry)
Right	Complex	Ischemia	NZ: 100% IC: 0.5% BZ: geometry		Fig 8		No (wave break/alternating CB)
Center					S3 Fig		
**Effect of EpC and myocardial ischemia on arrhythmia**							
Right	Complex	Ischemia	NZ: 100% IC: 0.5% BZ: geometry	Homogeneous (*d*_cleft_ = 8 nm)	Fig 9	Arrhythmia 1	Yes
Center					S4 Fig		
Right				Heterogeneous	Fig 10		Yes (longer time)
Center					S5 Fig		

## Materials and methods

### Modeling EpC

**Fig 1:**
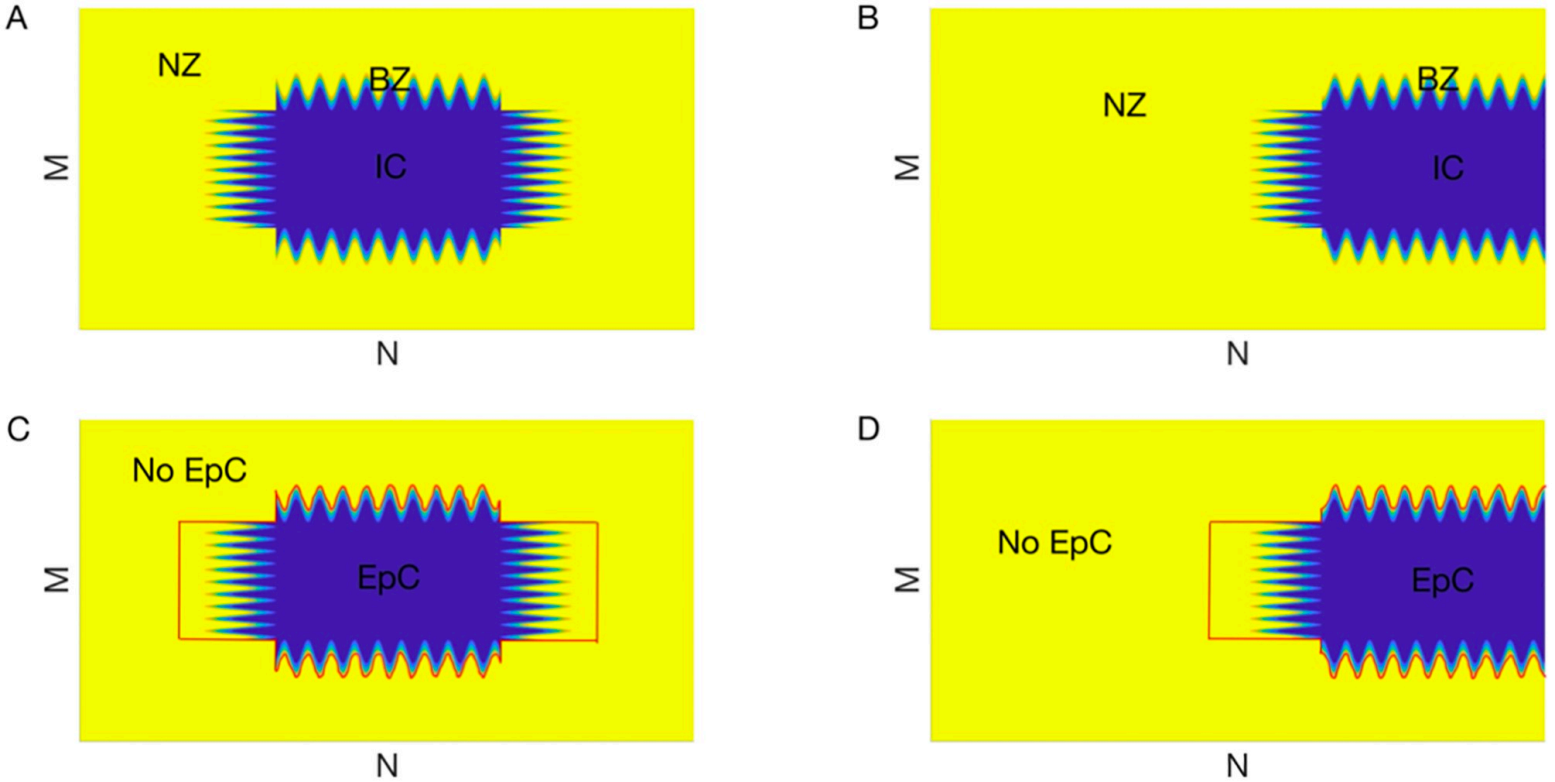
Two different locations of ischemic core (IC, blue) with complex geometry of border zone (BZ). (A) in the middle; and (B) on the right hand side of the lattice. Normal zone (NZ) is shown in yellow. Heterogeneous EpC is applied in the regions enclosed by red curves in (C) and (D).

We used a two-dimensional (2D) discrete microscopic bidomain model [14, 15] consisting of a large number of coupled, nonlinear, ordinary differential equations and algebraic equations, in which EpC was incorporated to model ephaptic conduction during acute myocardial ischemia. We previously reported (see Fig 1 in [14]) that there are four different compartments in our model, namely intracellular space, extracellular space, cleft space and extracellular cleft space. The model equations are set up based on the current conservation for each compartment (see [14] for details). The following cleft width: *d*_cleft_ = 2 nm, 5 nm, 8 nm, 13 nm, 15 nm and 115 nm were chosen based on [14, 15] as representative values of EpC in our numerical simulations. Note that smaller *d*_cleft_ results in large cleft resistance, which implies stronger EpC; in contrast, *d*_cleft_ = 115 nm indicates a near absence of EpC. The following distributions of EpC were considered: (1) homogeneous distribution, in which EpC is applied uniformly across the lattice; (2) heterogeneous distribution, in which EpC is only applied in IC and BZ; and (3) heterogeneous distribution, in which EpC is applied in both IC, BZ and part of NZ due to possible different *W*_*p*_ in NZ and ischemic region [19]. Heterogeneous distribution of EpC for the case (3) is shown in Fig 1C and 1D, for both locations of IC.

### Modeling acute regional myocardial ischemia

#### Complex geometry of BZ

We followed our experimental optical coherence tomography (OCT) findings [22] to model the “finger-like” geometry of BZ. As indicated in Fig 1, we considered two different geometries, in which the following three regions are incorporated: NZ (yellow), BZ (“fingers”) and IC (blue) with IC being located either in the center (A) or at the right hand side (B) of a *M* by *N* lattice (*M* = *N* = 550). BZ was modeled as the region where the IC protrudes into NZ with a complex “finger-like” structure (similar to [15]). We used the following parameters to model the shape of the BZ: *b*, *n*, and *L* for frequency, depth, and width of the “fingers” respectively; and *M*_IC_ × *N*_IC_ for IC size. The following configurations were incorporated for BZ in the center: *b* = 0.5, *n* = 30, *L* = 50, *M*_IC_ = 50, *N*_IC_; and for BZ on the right: *b* = 0.5, *n* = 10, *L* = 30, *M*_IC_ = 200, *N*_IC_ = 100.

#### Electrophysiological changes during myocardial ischemia

To represent the excitable cell dynamics in NZ, we adopted the Luo-Rudy dynamic (LRd) guinea pig ventricular model [23]. All ionic channels (except the fast Na^+^ channel) in the LRd model are uniformly distributed along the cell membrane. However, Na^+^ channels are localized to the cleft spaces between cell ends, while the total conductance of Na^+^ channels is fixed to its normal value [7, 14, 15].

We then followed the approaches of [15, 24, 25] to model the four main components of 10–15 min regional acute ischemia in IC: hyperkalaemia, acidosis, anoxia and gap junction remodeling. Hyperkalaemia was modeled by increasing the extracellular potassium concentration [K^+^]_o_ from 4.5 mM (NZ) to 14.5 mM (IC). The effects of acidosis were modeled in IC through a decrease in both the conductances of sodium (g_Na_) and L-type calcium (g_Ca_) channels by 25%; and through a positive voltage shift (3.4 mV) in the fast Na^+^ current kinetics. The direct electrophysiological effects of anoxia were modeled by reducing the intracellular Adenosine Triphosphate (ATP) concentration from 10 mM (NZ) to 3 mM (IC), which affects the ATP-dependent potassium and L-type calcium current. Gap junction remodeling was included as electrical uncoupling in IC and represented by a 99.5% reduction in connexin 43 (Cx43) expressed gap junctions, whereas 100% of gap junctions remain in NZ region. Similar to [15], we modeled all parameter values in BZ along the following curve *f*:

$$\begin{array}{c} f\left(x,y\right)=f_{0}\left(x-n\;\text{sin}\;by\right), \end{array}$$ (1)

where $f_{0}\left(z\right)=\frac{a_{\text{NZ}}+a_{\text{IC}}}{2}+\frac{a_{\text{NZ}}-a_{\text{IC}}}{2}\text{tanh}\left(\frac{z+L/2}{L/2}\right)$, *a*_NZ_ and *a*_IC_ represent the parameter values in NZ and IC, respectively. The relevant electrophysiological parameter values during myocardial ischemia are summarized in Table 2.

**Table 2:** Electrophysiological parameter changes during myocardial ischemia.

Parameter	Value	Reference	Impact
Extracellular potassium concentration ([K^+^]_o_)	4.5 mM (NZ)	[24, 25]	–
	14.5 mM (IC)	[24, 25]	hyperkalaemia
Conductance of sodium (g_Na_)	100% (NZ)	[24, 25]	–
	75% (IC)	[24, 25]	acidosis
Conductance of calcium (g_Ca_)	100% (NZ)	[24, 25]	–
	75% (IC)	[24, 25]	acidosis
A voltage shift in fast Na^+^ kinetics	0 mV (NZ)	[24, 25]	–
	3.4 mV (IC)	[24, 25]	acidosis
ATP concentration ([ATP]_i_)	10 mM (NZ)	[24, 25]	–
	3 mM (IC)	[24, 25]	anoxia
Gap junctional remodeling	100% (NZ)	[15, 24, 25]	–
	0.5% (IC)	[15]	electrical uncoupling

### Numerical simulation

The action potential propagation can be remarkably slow and discontinuous in ischemic cardiac tissue, and the potentials and ionic concentrations of a cell can thus be very different from its neighbors. Therefore, our discretized model introduced in [14, 15] can more precisely capture the discontinuous characteristics of cardiac propagation. The wavefront of normal action potential propagation was defined as the spatial location where the side transmembrane potential (*V*_*m*_) exceeds −30 mV and $\frac{\partial V_{m}}{\partial t}>0$. This value was set as a threshold to distinguish normal action potential from small amplitude action potential (SAP) [14, 15]. SAP was defined in [14] as a novel type of action potential that can propagate in the cardiac tissue despite its low amplitude (maximum V_*m*_ = −40 mV).

We run numerical simulations in a lattice with *M* × *N* cells, where *M* = 550 and *N* = 550 with a time step of 0.01 ms. In order to generate a reentry in the 2D tissue, we applied an S1-S2 cross-field stimulation protocol, with the first stimulus (S1) being delivered to the left boundary of the lattice at *t* = 0 with amplitude of 0.15*μ*A and a duration of 2 ms. The second stimulus (S2) was delivered to the bottom of the lattice at variable S1S2 intervals of the same amplitude and duration. Activation was monitored by plotting *V*_*m*_ at different location and time snapshots. Initially, all gating variables, ionic concentrations and potentials were set at steady state. We used a splitting method to update the potential, ionic concentration and gating variables of ion channels separately to solve this system. Specifically, the linear parts of the system were treated using backward Euler method, and the nonlinear parts (e.g. the ionic currents and dynamics) were linearized first and then treated using backward Euler method. The system was solved via a direct method (backslash operator in Matlab).

## Results

### The effects of EpC on reentry in the absence of myocardial ischemia

#### The impact of homogeneous EpC on reentry in NZ

**Fig 2:**
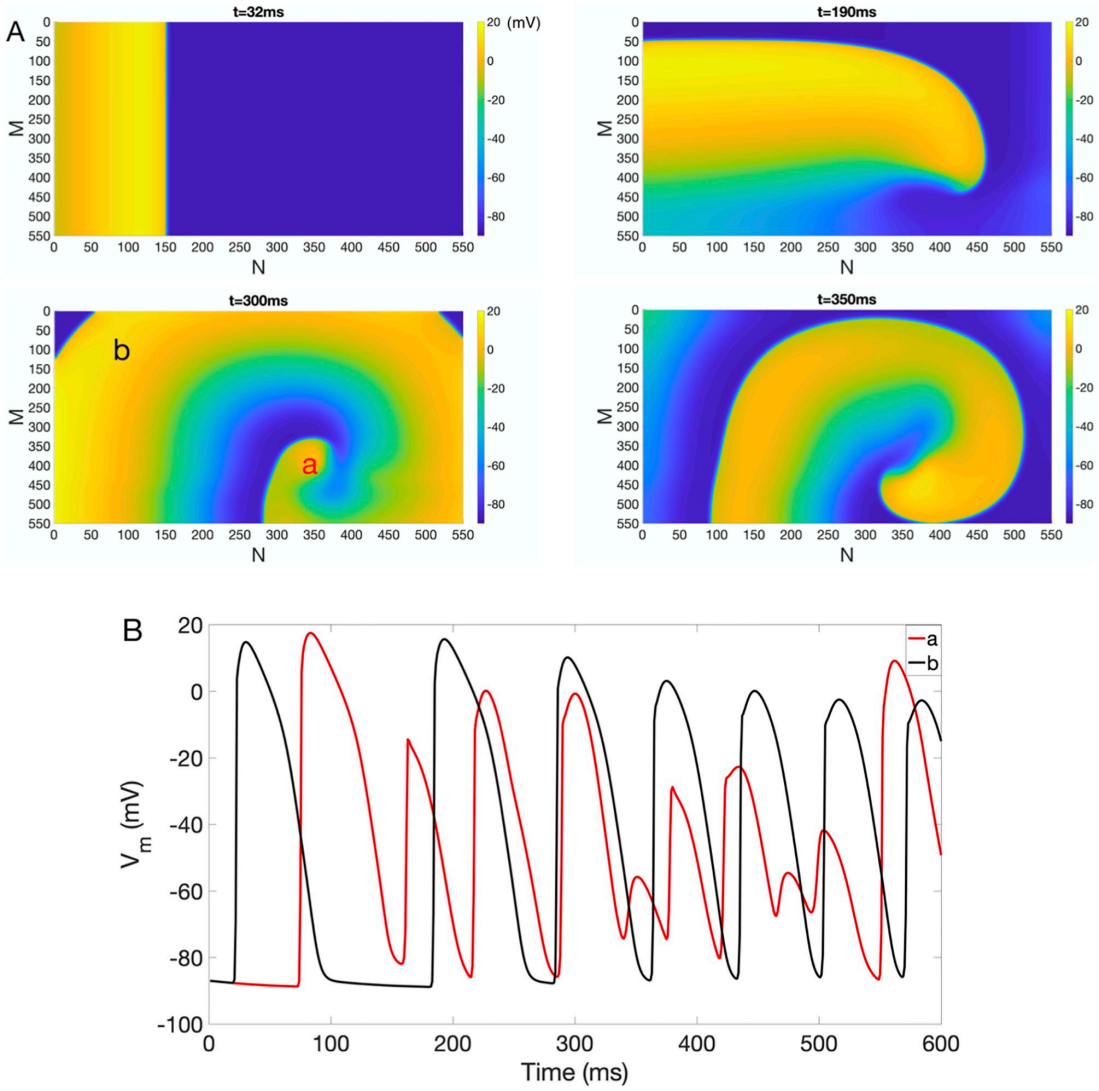
Reentry generated in NZ in the absence of EpC. (A) Reentry generated in 2D normal tissue consisting of 550 by 550 cells in the absence of EpC (*d*_cleft_ = 115 nm). Colorbar indicates *V*_*m*_ (in the units of mV). Snapshots of *V*_*m*_ at time = 32 ms, 190 ms, 300 ms and 350 ms are shown. (B) Representative *V*_*m*_ traces for points close to the core of the reentry (*a*, red) and at the periphery (*b*, black).

We first generated a *self-sustaining* reentry in the absence of EpC (*d*_cleft_ = 115 nm) in normal tissue by applying an S1-S2 cross-field stimulation protocol with S1S2 = 140 ms. Fig 2A demonstrates snapshots of *V*_*m*_ during a sustained reentry in NZ at *t* = 32 ms, 190 ms, 300 ms and 350 ms. Fig 2B shows representative traces of *V*_*m*_ close to the center of the reentry (*a*) and at the periphery (*b*). As indicated in Fig 2B, *V*_*m*_ close to the core of the reentry expresses very rapid and chaotic behavior, in contrast to the fast but regular activity at the periphery.

**Fig 3:**
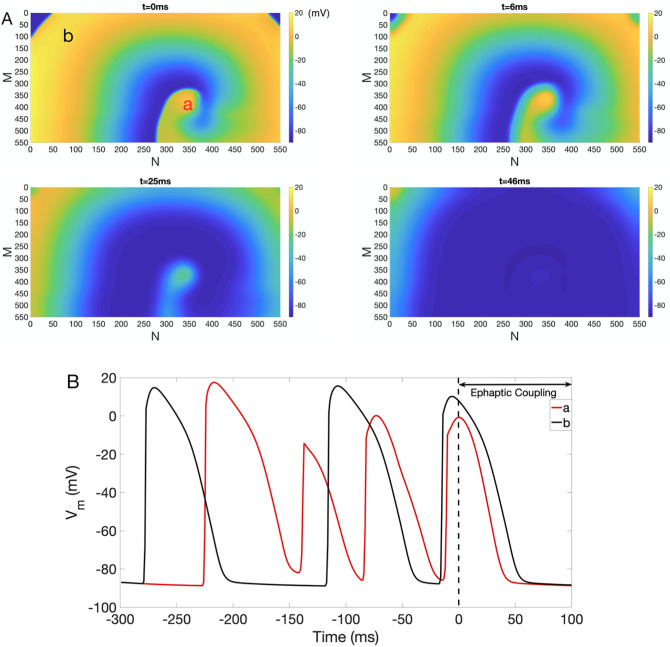
Inhibition of reentry in NZ under homogeneous EpC. (A) Suppression of reentry in the presence of homogeneous EpC at *d*_cleft_ = 8 nm. Colorbar indicates *V*_*m*_ (in the units of mV). Snapshots of *V*_*m*_ at time = 0 ms, 6 ms, 25 ms and 46 ms are shown. (B) Representative *V*_*m*_ traces at points *a* (red) and *b* (black).

To explore the impact of homogeneous EpC on the evolution of reentry in NZ, we used this self-sustained reentry as initial conditions to generate Fig 3, S1 and S2 Figs to ensure that our results are independent of reentry initiation methods. We used the snapshot of *t* = 300 ms of Fig 2A as initial condition (*t* = 0) and incorporated EpC of different strength (various *d*_cleft_) to investigate its effect on dynamics of arrhythmia and its termination. As an example, Fig 3A shows snapshots of *V*_*m*_ at *t* = 0 ms, 6 ms, 25 ms and 46 ms in the presence of homogeneous EpC at *d*_cleft_ = 8nm. As indicated in Fig 3A, reentry is suppressed in the presence of EpC (see representative traces of V_*m*_ in Fig 3B), which points to an anti-arrhythmic effect of EpC. S1 and S2 Figs demonstrate that weaker homogeneous EpC (*d*_cleft_ 13 nm and 23 nm, respectively) has similar effect. Specifically, arrhythmia is successfully terminated in both cases, but it takes longer time for weaker EpC to successfully terminate arrhythmia.

#### The impact of homogeneous EpC on reentry in poorly coupled tissue

**Fig 4:**
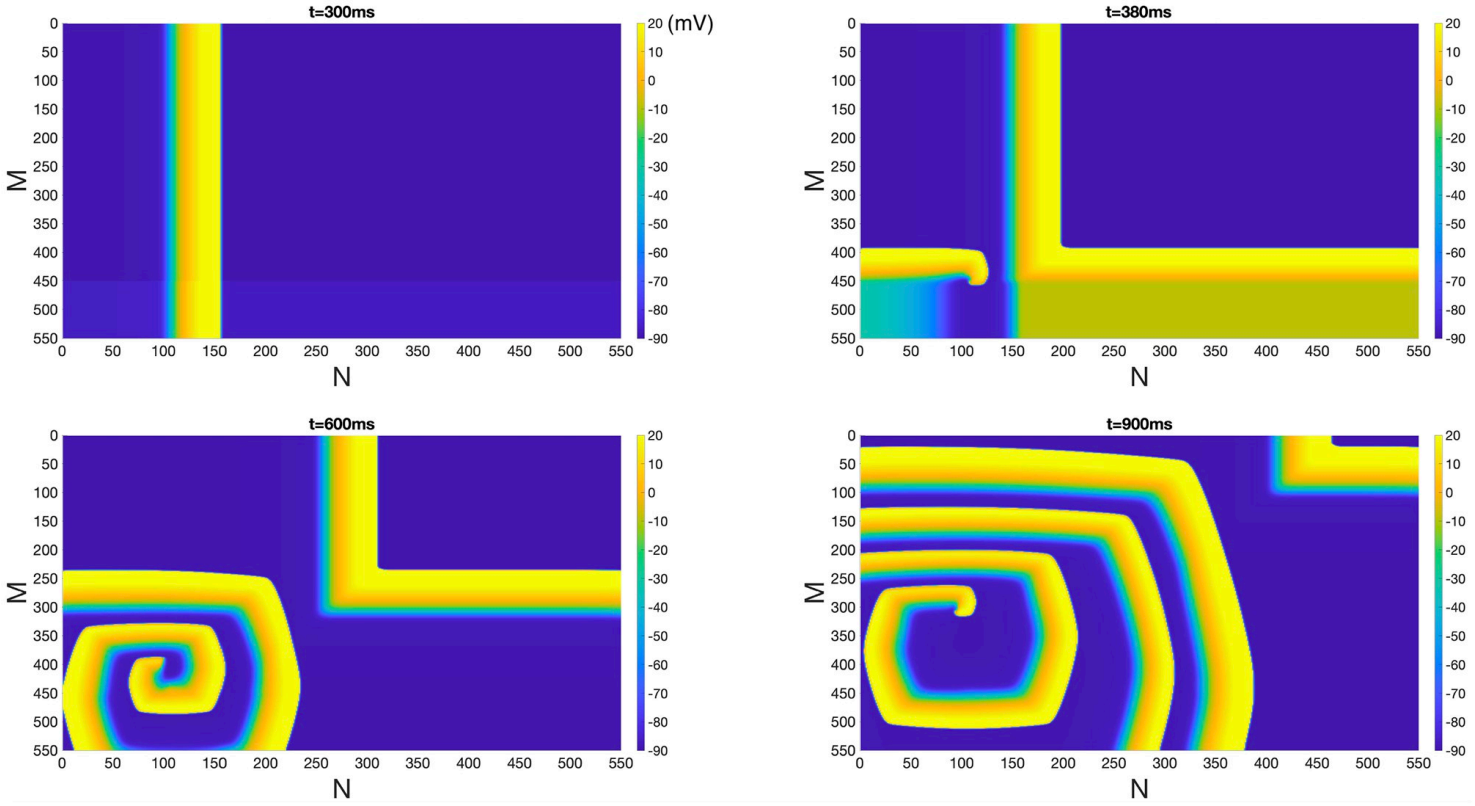
Reentry generated in a poorly coupled tissue in the absence of EpC. Reentry generated in 2D tissue of low gap junctions (1% of gap junctional coupling remains) of 550 by 550 cells in the absence of EpC. Colorbar indicates *V*_*m*_ (in the units of mV). Snapshots of *V*_*m*_ at time = 300 ms, 380 ms, 600 ms and 900 ms are shown.

We then generated a *self-sustaining* reentry in the absence of EpC (*d*_cleft_ = 115 nm) in the poorly coupled tissue, where only 1% of gap junction remains. Fig 4 shows the snapshots of *V*_*m*_ at *t* = 300 ms, 380 ms, 600 ms and 900 ms. Note that a narrower width of the wavefront is due to slower CV and smaller action potential duration (APD) when compared to the results of Fig 2.

**Fig 5:**
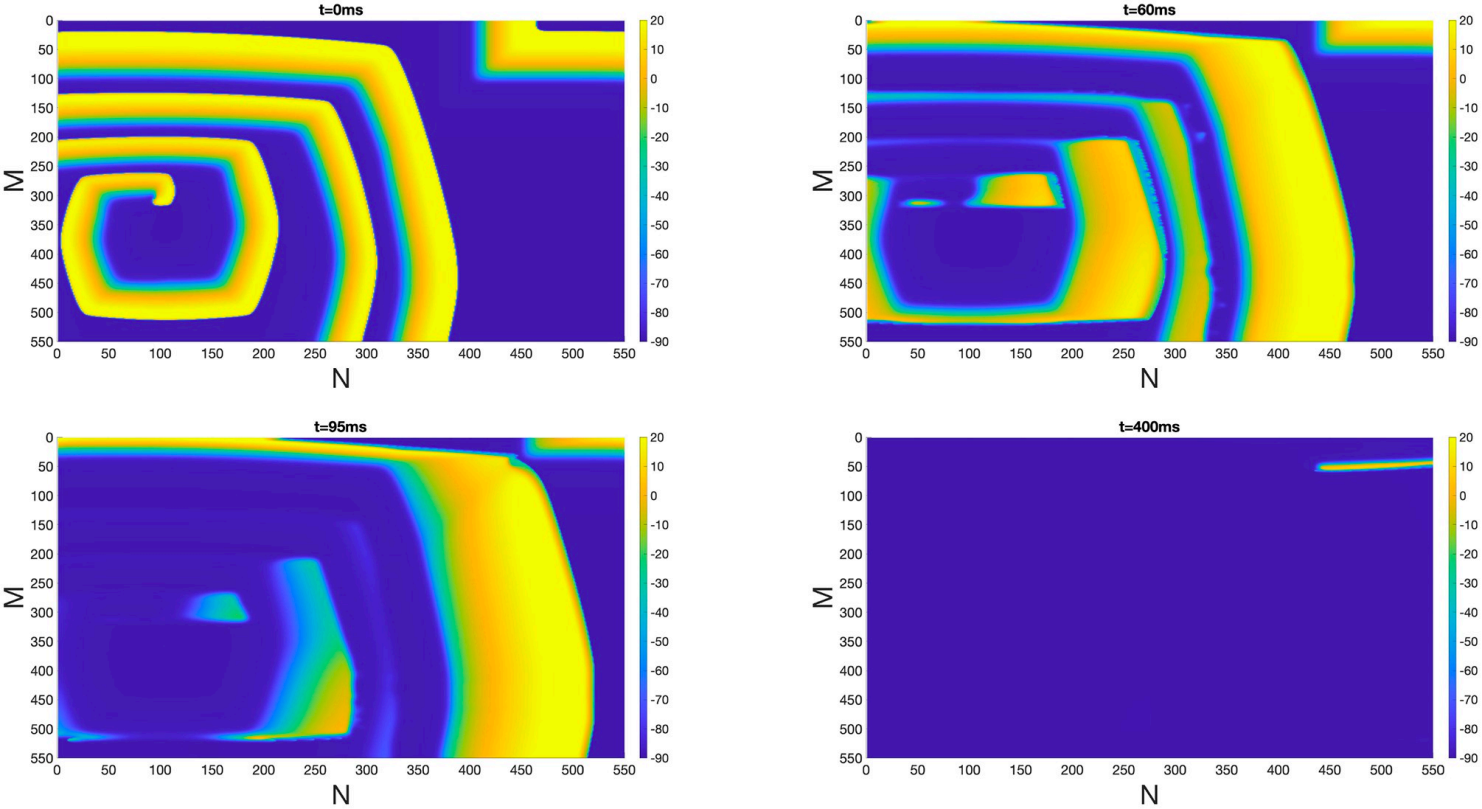
Inhibition of reentry in a poorly coupled tissue under homogeneous EpC. Suppression of reentry generated in poorly coupled tissue in the presence of homogeneous EpC at *d*_cleft_ = 8 nm. Colorbar indicates *V*_*m*_ (in the unit of mV). Snapshots of *V*_*m*_ at time = 0 ms, 60 ms, 95 ms and 400 ms are shown.

To explore the impact of homogeneous EpC on the evolution of reentry, we used the snapshot of *t* = 900 ms of Fig 4 as initial condition (*t* = 0) and applied EpC at *d*_cleft_ = 8 nm. Fig 5 shows snapshots of *V*_*m*_ at *t* = 0 ms, 60 ms, 95 ms and 400 ms, which indicates a successful suppression of reentry, similar to the effect observed in normal tissue (Fig 3, S1 and S2 Figs).

### The effects of myocardial ischemia on reentry

#### The impact of physiological components of myocardial ischemia on reentry

We then explored the impacts of individual components of acute myocardial ischemia (hyperkalaemia, acidosis, anoxia and electrical uncoupling) on the evolution of reentry in the absence of EpC. For this, we used the snapshot of *t* = 300 ms of Fig 2A as initial condition (*t* = 0) and incorporated each component of ischemia separately to study their role on the evolution of reentry.

**Fig 6:**
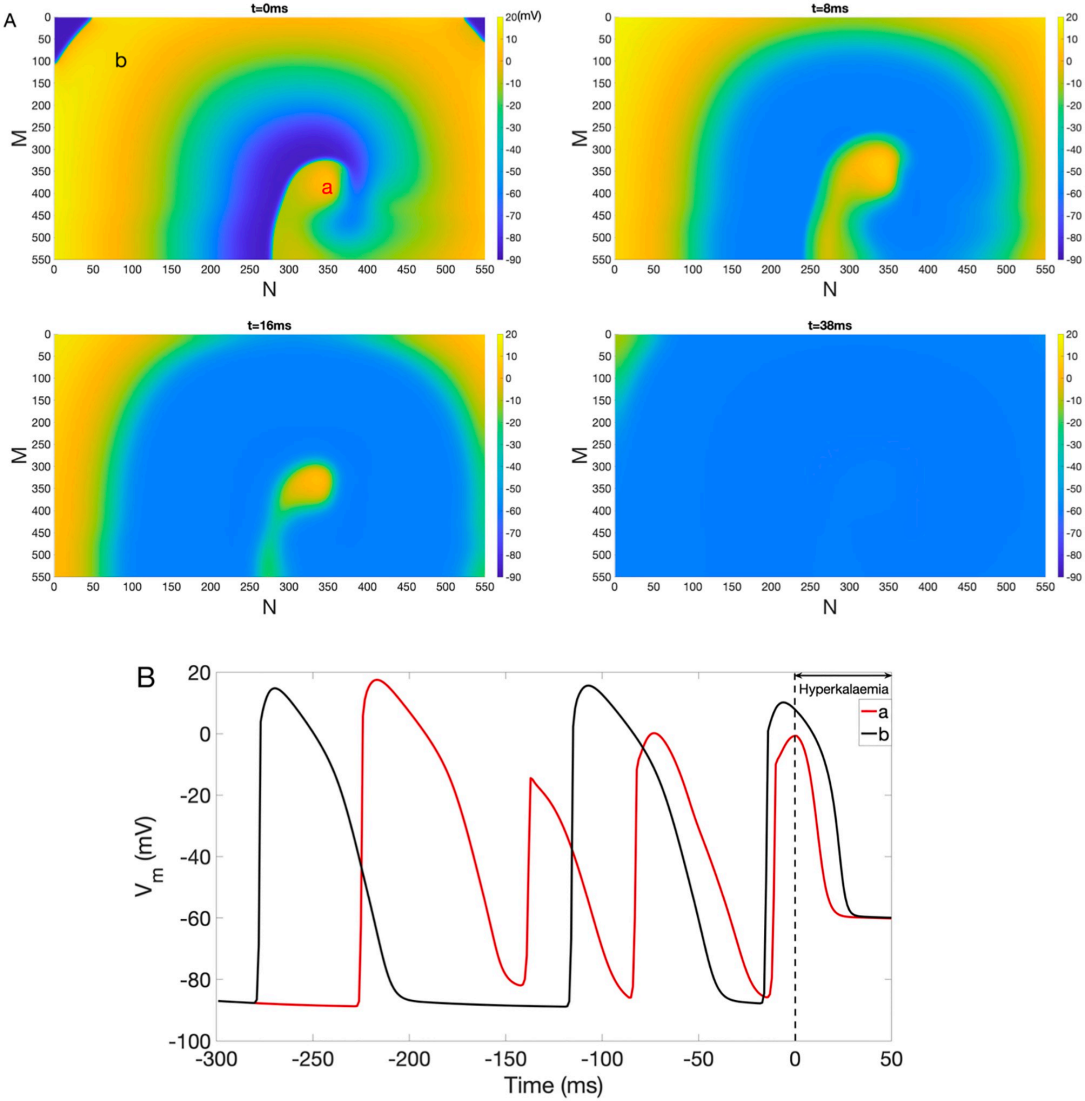
Inhibition of reentry under hyperkalaemia. (A) Suppression of reentry under the hyperkalaemia condition. Colorbar indicates *V*_*m*_ (in the unit of mV). Snapshots of *V*_*m*_ at time = 0 ms, 8 ms, 16 ms and 38 ms are shown. (B) Representative *V*_*m*_ traces at point *a* (red) and *b* (black).

The effect of hyperkalaemia on reentry is shown in Fig 6A, which plots snapshots of *V*_*m*_ at *t* = 0 ms, 8 ms, 16 ms and 38 ms and demonstrates the termination of reentry (see representative traces of *V*_*m*_ in Fig 6).

**Fig 7:**
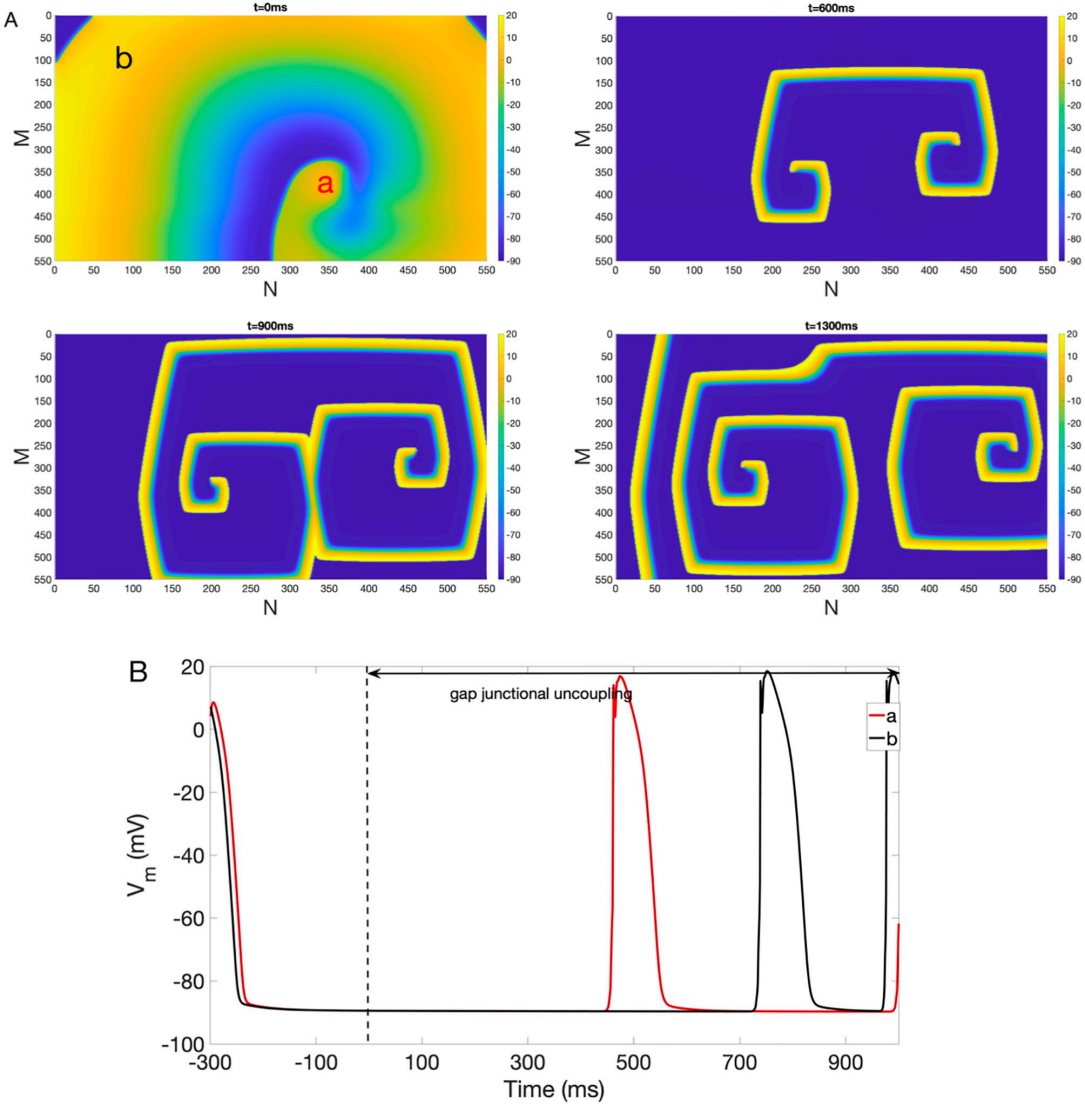
Multiple spiral waves generated under gap junctional uncoupling. (A) Multiple reentry in the presence of gap junctional uncoupling. Colorbar indicates *V*_*m*_ in the unit of mV. Snapshots of *V*_*m*_ at time = 0 ms, 600 ms, 900 ms and 1300 ms are shown. (B) Representative *V*_*m*_ traces at point *a* (red) and *b* (black).

In contrast, acidosis and anoxia preserve reentry and its dynamics. Interestingly, gap junctional uncoupling/remodeling preserves reentry but affect its dynamics. For example, Fig 7A plots snapshots of *V*_*m*_ at *t* = 0 ms, 600 ms, 900 ms and 1300 ms under gap junctional uncoupling alone. As indicated in Fig 7A, multiple waves are born and collide a narrower wavefront, which results from a shorter APD (Fig 7B) and a slower CV. Fig 7B shows *V*_*m*_ traces at point *a* and *b* indicating different properties of action potential as a results of different spatio-temporal dynamics of reentry under gap junctional uncoupling.

#### The impact of complex geometry of BZ on reentry

We further explored the impact of BZ geometry on reentry evolution in the absence of EpC. Note that when IC BZ is located in the center of the lattice, the core of reentry shown in Fig 2A is closer to IC BZ at *t* = 350 ms than at *t* = 300 ms. We therefore used the snapshots of *t* = 300 ms and 350 ms of Fig 2A as initial conditions (*t* = 0) when IC BZ is located on the right and in the center of the lattice, respectively.

**Fig 8:**
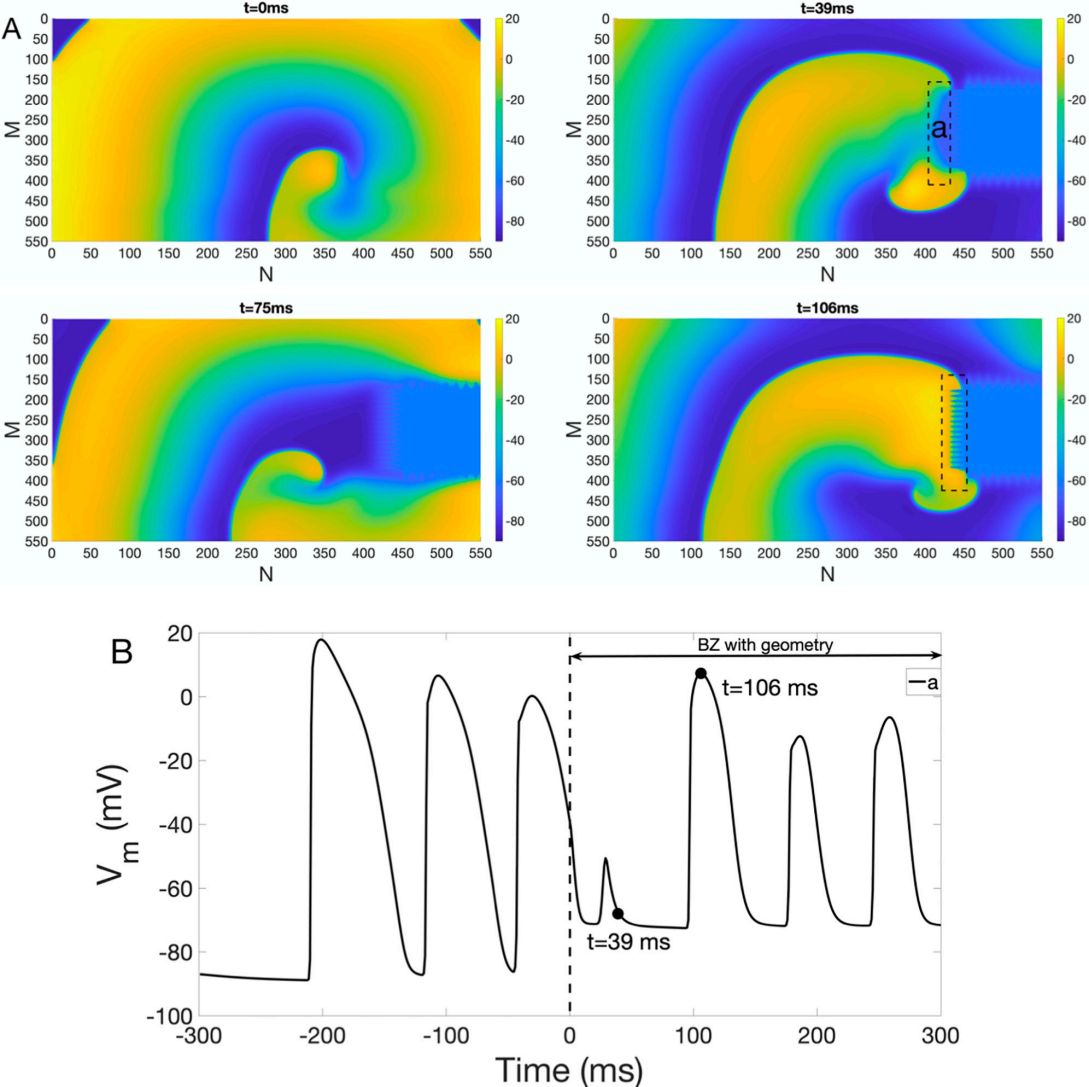
Spiral wavebreak and alternating CB under IC BZ with complex geometry (right). (A) Spiral wave break-up and alternating CB in the presence of BZ with complex geometry on the right. Colorbar indicates *V*_*m*_ (in the unit of mV). Snapshots of *V*_*m*_ at time = 0 ms, 39 ms, 75 ms and 106 ms are shown. Dashed black box indicates the place where wave break occurs. (B) *V*_*m*_ traces at point *a*.

Fig 8A shows snapshots of *V*_*m*_ at *t* = 0 ms, 39 ms, 75 ms and 106 ms, where IC with complex BZ geometry is located on the right hand side of the lattice. As indicated in Fig 8A, spiral wave break-up and 2:1 alternating conduction block (CB) are seen as the wavefront getting close to the IC BZ region. Here 2:1 alternating CB is the conduction failure occurring on alternate beats. For example, one can see that wave break-up occurs *away* from the IC BZ region at *t* = 39 ms. However, wavebreaks occurs *next to* IC BZ region at *t* = 106 ms, which causes alternating CB. Alternating CB and wave break-up point to a pro-arrhythmic role of BZ with a complex geometry. Fig 8B shows action potential trace for a point *a* in the BZ. The voltage trace is a representative example of alternating CB, where CB occurs at *t* = 39 ms, but successful propagation is seen at *t* = 106 ms.

Similar to Fig 8A, alternating CB and wave break-up are also seen in S3A Fig, where IC BZ is located in the center of the lattice. S3B Fig shows the action potential trace for a point *a* in the BZ. The voltage trace is also indirectly demonstrate the presence of alternating CB, in which CB occurs at *t* = 31 ms, but successful conduction is seen at *t* = 105 ms.

### The effect of EpC on reentry in the presence of BZ with complex geometry

#### Homogeneous EpC

Results in the previous sections suggest that homogeneous EpC and BZ with complex geometry have opposite effects on the reentry. In particular, the presence of homogeneous EpC terminates reentry (see Fig 3, S1 and S2 Figs), whereas BZ with complex geometry alone facilitates reentry by producing wave break-up and alternating CB (Fig 8 and S3 Fig).

**Fig 9:**
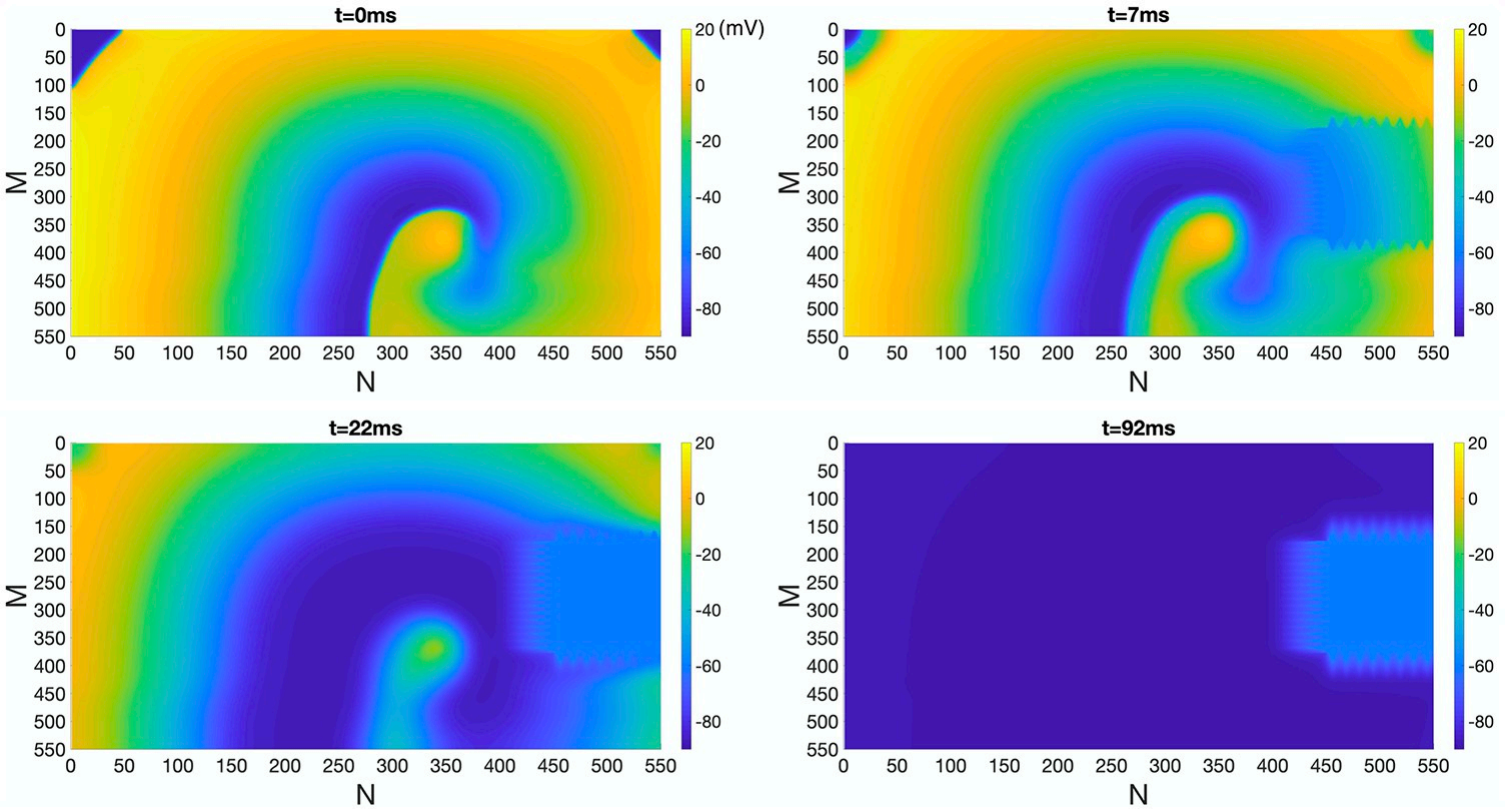
Inhibition of reentry under homogeneous EpC and IC BZ with complex geometry (right). Suppression of reentry in the presence of homogeneous EpC (*d*_cleft_ = 8 nm) and BZ with complex geometry to the right of the lattice. Colorbar indicates *V*_*m*_ (in the unit of mV). Snapshots of *V*_*m*_ at time = 0 ms, 7 ms, 22 ms and 92 ms are shown.

Here, we aim to investigate the interplay of both factors on the evolution of reentry. Similar to numerical simulation performed in the previous sections, we used the snapshots of *t* = 300 ms and 350 ms of reentry in Fig 2A as initial conditions, when IC BZ is located on the right and in the center of the lattice, respectively. We then performed numerical simulations in the presence of homogeneous EpC and BZ with complex geometry. Fig 9 plots snapshots of *V*_*m*_ at *t* = 0 ms, 7 ms, 22 ms and 92 ms at *d*_cleft_ = 8 nm. As indicated in Fig 9 and S4 Fig, reentry is terminated in the presence of homogeneous EpC despite the presence of complex geometry of the IC BZ, independent of the location of IC BZ. We further investigated the effect of different strength of EpC (by varying d_cleft_ from 8 nm to 30 nm) on the reentry termination. Our results (not shown) demonstrate that weaker EpC takes longer time to terminate reentry in the presence of complex geometry of IC BZ.

#### Heterogeneous EpC

The EpC is highly dependent on the cleft width (*d*_cleft_). Since the reduction in gap junctional coupling and cell swelling are both heterogeneous in the heart during myocardial ischemia, we then explored the interplay between *heterogeneous* EpC and BZ with complex geometry on the termination of reentry. Specifically, we considered the following two scenarios: (1) EpC is present in both IC BZ and part of NZ (see panels C and D of Fig 1) or (2) EpC is only present in IC BZ.

**Fig 10:**
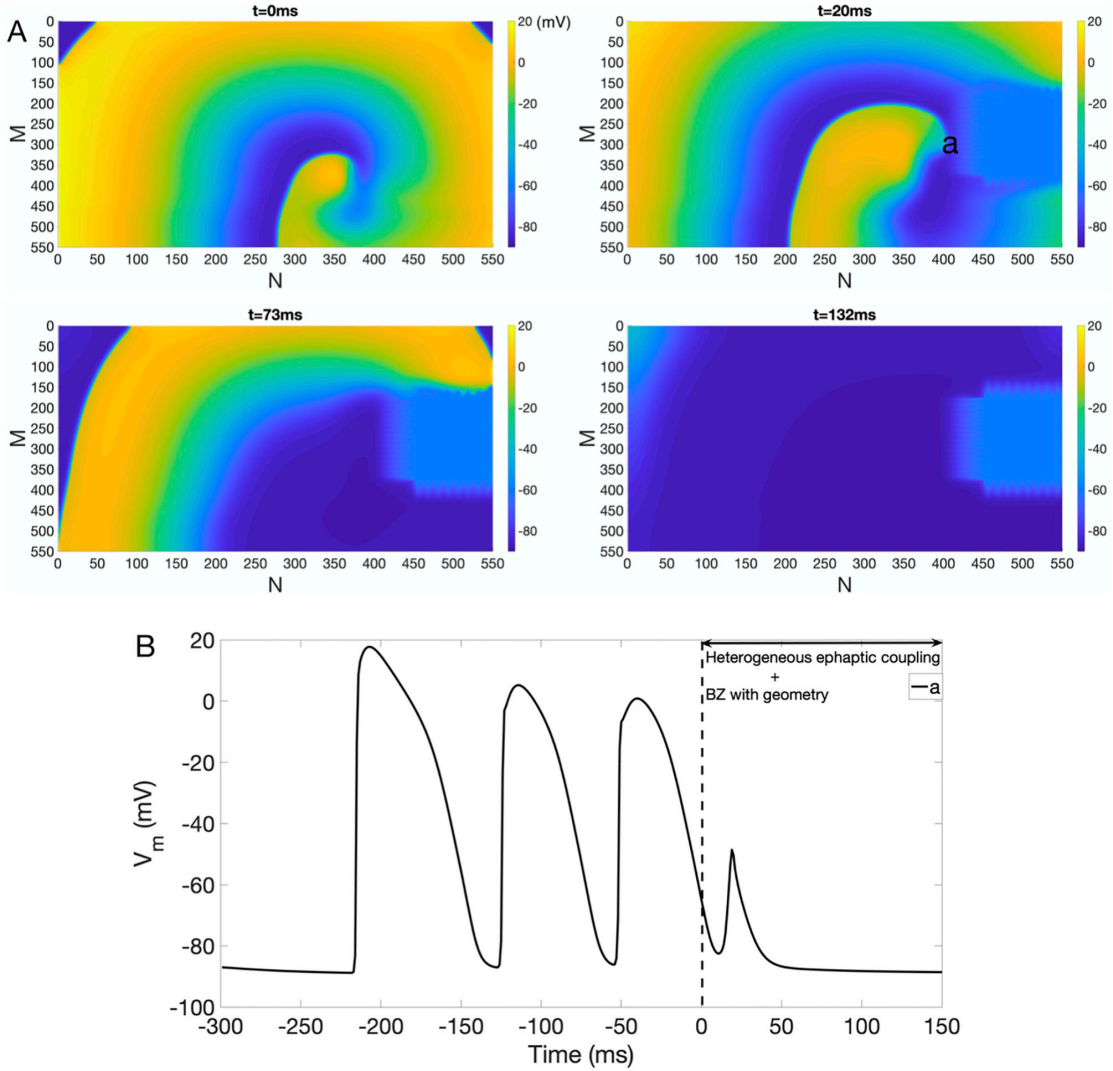
Inhibition of reentry under heterogeneous EpC and IC BZ with complex geometry (right). (A) Suppression of reentry in the presence of EpC present in IC BZ and part of NZ and BZ geometry on the right of the lattice. Colorbar indicates *V*_*m*_ (in the unit of mV). Snapshots of *V*_*m*_ at time = 0 ms, 20 ms, 73 ms and 132 ms are shown. (B)*V*_*m*_ trace of point *a*.

To simulate scenario (1), we chose *d*_cleft_ = 13 nm in NZ enclosed by the red curve, *d*_cleft_ = 2 nm in IC, and *d*_cleft_ values in BZ were calculated according to Eq 1. Similar to previous sections, we used the snapshots of *t* = 300 ms and 350 ms of reentry in Fig 2A as initial conditions when IC BZ is located on the right and in the center of the lattice, respectively. Then we performed numerical simulations for two different locations of IC: on the right (Fig 10) and in the middle (S5 Fig) of the lattice. Fig 10A plots snapshots of *V*_*m*_ at *t* = 0 ms, 20 ms, 73 ms and 132 ms, respectively. As indicated in Fig 10A, the reentry is suppressed in the presence of heterogeneous EpC, despite the presence of BZ with complex geometry. To explain the mechanism of reentry termination, we plot the action potential trace for a point *a* (see Fig 10B). As shown in Fig 10B, *V*_*m*_ at point *a* rises to −50 mV around 20 ms after the presence of heterogeneous EpC and BZ with complex geometry. However, the inward Na^+^ current is not sufficient to sustain the full depolarization, transmembrane potential of point *a* thus returns to steady state afterwards.

S5A Fig plots snapshots of *V*_*m*_ at *t* = 0 ms, 52 ms, 300 ms and 457 ms. As indicated, the reentry can also be suppressed in the presence of heterogeneous EpC, despite the presence of BZ with complex geometry in the center. S5B Fig plots *V*_*m*_ trace for a point *a*. As shown in S5B Fig, peak of *V*_*m*_ at point *a* gradually decreases from 10 mV to −90 mV (along the damped oscillation) with the combination of heterogeneous EpC and BZ with complex geometry. The upstroke of *V*_*m*_ occurred at 350 ms demonstrates a planar wave propagation. Unlike Fig 10, the central location of BZ provides a larger number of pathways for the wavefront to bypass the low excitability region. Therefore, it takes longer to suppress the reentry. Our results also show that reentry cannot be terminated for scenario (2) when EpC is *only* present in IC BZ.

## Discussion

This is by far the *first* study to explore the interplay among EpC, anatomical and electrophysioloigical gradient developed at IC BZ on the termination of reentry. Our results shows that EpC can inhibit reentry under non-ischemic and ischemic conditions, which is the *first* instance to theoretically show the anti-arrhythmic role of EpC. In order to ensure that the impact of EpC on reentry termination is independent of pacing protocol and initial condition, we therefore used one snapshot of a sustained reentry generated in NZ and poorly coupled tissue, respectively as an initial condition and then applied EpC.

Although the exact mechanism behind the inhibition of reentry in the presence of EpC is still unknown and needs further investigation, we suggest that a decrease in the activating current or an increase an in the electrical loads during myocardial ischemia might be important players. Specifically, in the presence of EpC, the high resistance between cleft space and extracellular space results in a negative cleft potential and consequently a large overshoot of *V*_*m*_, which results in a self-attenuation of the driving force of localized *source current* (I_Na_) to the cleft for cells downstream. In addition, in the presence of BZ with complex geometry, as the action potential propagates (during reentry) from poorly ephaptically coupled region (i.e. NZ) to well ephaptically coupled region (i.e. IC BZ), a large activation current is required to excite cells in IC BZ. The presence of heterogeneous EpC may thus significantly increase the electric load. We therefore suggest that EpC contributes to the termination of reentry either by decreased activating current or increased electrical loads. Note that after reentry is inhibited, the tissue can still be excited by another stimulus. However, the same mechanism can potentially explain the proarrhythmic role of EpC in a different scenario, such as reentry initiation, which is a topic of our undergoing study.

Heterogeneities and electrophysiological gradients developed at IC BZ are pro-arrhythmic [1–3] due to increased occurrence of unidirectional block and reentry arrhythmia [3, 26]. IC BZ is thus commonly known to be one of the prominent substrates for lethal ventricular arrhythmias [1, 27]. IC BZ were incorporated in several conventional models of cardiac electrophysiology [3, 24, 28, 29]. For example, [28] is a leading review paper discussed recent advances in computational medicine, with specific examples in the fields of cancer, diabetes, cardiology, and neurology. The paper [29] cited in the [28] mentioned the combination of periinfarct zone (PZ) ionic current remodeling and different degrees of myofibroblasts (Mfb) infiltration in the infarcted ventricles determines susceptibility to arrhythmia. In particular, Mfbs exacerbate arrhythmia propensity at intermediate densities; In contrast, Mfbs can overcome the arrhythmogenic effects of PZ ionic current remodeling at high densities. However, a significant omission in these models is the anatomical changes occurred in IC BZ, which limits the ability to explore the role of IC BZ in arrhythmia termination. We are the *first* to take electrophysiological and anatomical gradients into account.

In order to get a comprehensive understanding of the impact of IC BZ geometry on reentry termination, we performed an additional set of simulations as follows: (1) BZ is present on the right hand side of the lattice but does not have a “finger-like” geometry (*n* = 0, *L* is the same as Fig 8) and (2) varying parameters *b*, *n* and *L*, one at a time based on Fig 8. For (1), our results show that the development of reentry is not significantly different from that of Fig 8. For (2), if only *b* is varied, the behavior of the propagating wave is not significantly different from that of Fig 8; however, if *n* or *L* becomes sufficiently large, then wave break and alternating conduction block (CB) are not seen. Instead, the propagating waves bypass the ischemic tissue. Therefore, we believe that the geometry of IC BZ play a more important role in reentry initiation instead of reentry termination.

As far as we know that there is no direct experimental evidence for the existences of EpC in the heart. Therefore, groups of mathematician and cardiologist attempt to validate the existence of EpC and reveal its physiological role and clinical relevance [6–17]. Studies of [6–15] show that EpC can either increase CV or reduce CB when gap junctions are compromised. However, some recent studies [16, 17] demonstrate that EpC is pro-arrhythmic. In particular, [16] shows that EpC is sensitive to the occurrence of phase 2 reentry for patients suffering from Brugada syndrome. In particular, phase 2 reentry is only seen when *W*_*p*_ is between 7 nm and 17 nm using 1D simulation. In [17], the authors show that the onset of arrhythmia is delayed by the intervention of EpC, which is determined by expansion of perinexal or a reduction in [Na]_o_. Based on the aftermentioned discussion, the detailed impact of EpC on arrhythmogesis is still unclear. Our study is highly interdisciplinary and provides a bridge between mathematical modeling and cardiac electrophysiology, which lays a solid ground for understanding and developing antiarrhythmic strategies and therapies for patients with structurally abnormal hearts (such as myocardial infarction), heart failure, or cardiomyopathy.

In our manuscript, we adopted a simplified discretized ordinary differential equation (ODE) model (see [14] for details) considering the discontinuous and slow action potential propagation in the ischemic heart, which makes cell potential and concentrations be very different from its neighbors. Therefore, using discretized ODE model allows us to better capture the discontinuous features. To gauge computational challenge posed by simulating the system with full microscopic model, we propose to build up a 2D multiscale model in the near future, which can be extended to three-dimensional.

One limitation of our study is that we adopted a simplified cell geometry, discretization (each cell is treated to be isopotential), and arrangement of cells. We believe that using realistic cell shape or cell arrangement will not qualitatively affect our results. The other limitation of our study is that we didn’t incorporate the fibrosis and its triggered activities in the IC BZ, which is an important aspect of ischemic region. We will work on this in the near future.

## Conclusion

In this manuscript, we explored the impact of EpC on reentry termination during non-ischemic and ischemic condition. Specifically, we investigated the contribution of EpC, electrophysiological and anatomical components of myocardial ischemia on reentry termination. Our main finding are listed as follows: (a) EpC terminates reentry initiated in NZ and poorly coupled tissue; it takes longer time for weaker EpC to complete termination. (b) Electrophysiological and anatomical components of ischemia affect evolution of reentry in the absence of EpC. In particular, hyperkalaemia terminates reentry; acidosis, anoxia and gap junctional uncoupling/remodeling preserve reentry, but affects its dynamics; BZ with complex geometry give rise to wave break and alternating CB. (c) Reentry is terminated in the presence of either homogeneous or heterogeneous EpC despite the presence of complex geometry of BZ. That effect is independent of the location of IC BZ. The inhibition of reentry can be attributed to a current-to-load mismatch. Our findings demonstrate a pro-arrhythmic role of BZ with complex geometry and an anti-arrhythmic role of EpC.

## Supporting information

S1 Fig Suppression of reentry in NZ under homogeneous EpC at *d*_cleft_ = 13 nm. Colorbar indicates *V*_*m*_ (in the units of mV). Snapshots of *V*_*m*_ at time = 0 ms, 20 ms, 207 ms and 438 ms are shown.
(PDF)

S2 Fig Suppression of reentry in NZ under homogeneous EpC at *d*_cleft_ = 23 nm. Colorbar indicates *V*_*m*_ (in the units of mV). Snapshots of *V*_*m*_ at time = 0 ms, 16 ms, 82 ms and 499 ms are shown.
(PDF)

S3 Fig Spiral wavebreak and alternating CB under IC BZ with complex geometry (center). (A) Spiral wave break-up and alternating CB in the presence of BZ with complex geometry in the center. Colorbar indicates *V*_*m*_ (in the unit of mV). Snapshots of *V*_*m*_ at time = 0 ms, 31 ms, 69 ms and 105 ms are shown. Dashed black box indicates the place where wave break occurs. (B) *V*_*m*_ trace of point *a*.
(PDF)

S4 Fig Inhibition of reentry under homogeneous EpC and IC BZ with complex geometry (center). Suppression of reentry in the presence of EpC (*d*_cleft_ = 8 nm) and BZ with complex geometry in the center of the lattice. Colorbar indicates *V*_*m*_ (in the unit of mV). Snapshots of *V*_*m*_ at time = 0 ms, 9 ms, 23 ms and 100 ms are shown.
(PDF)

S5 Fig Inhibition of reentry under heterogeneous EpC and IC BZ with complex geometry (center). (A) Suppression of reentry in the presence of EpC present in IC BZ and part of NZ and BZ geometry in the center of the lattice. Colorbar indicates *V*_*m*_ (in the unit of mV). Snapshots of *V*_*m*_ at time = 0 ms, 52 ms, 300 ms and 457 ms are shown. (B) *V*_*m*_ trace of point *a*.
(PDF)
