# Supplementary figures and images for: Mechanisms of arrhythmia termination during acute myocardial ischemia: Role of ephaptic coupling and complex geometry of border zone
Source: PLoS One. 2022 Mar 15;17(3):e0264570. doi: 10.1371/journal.pone.0264570 (PMC8923475; doi:10.1371/journal.pone.0264570)

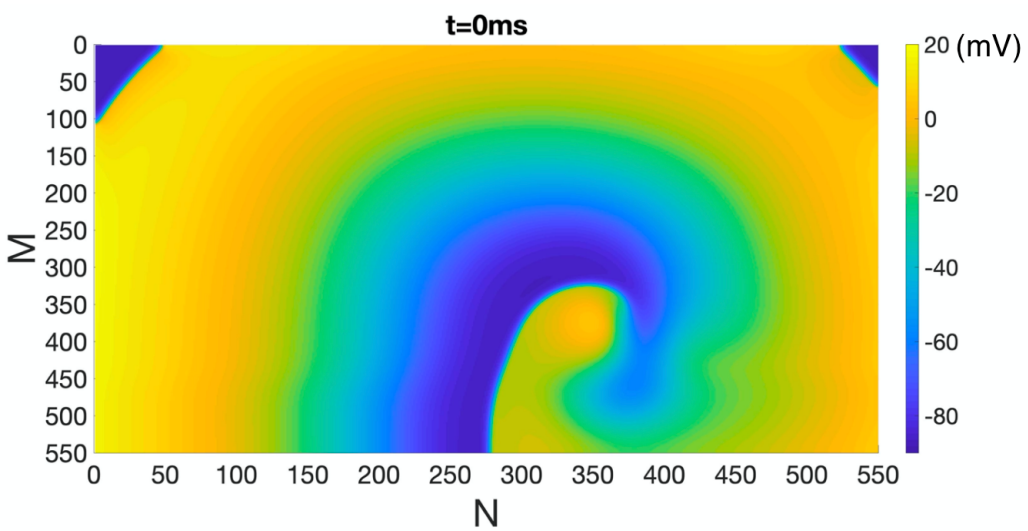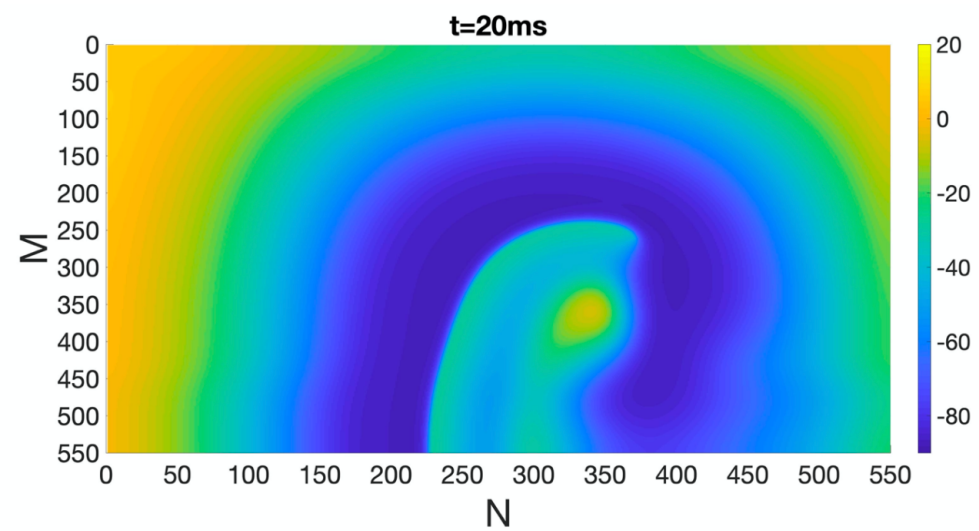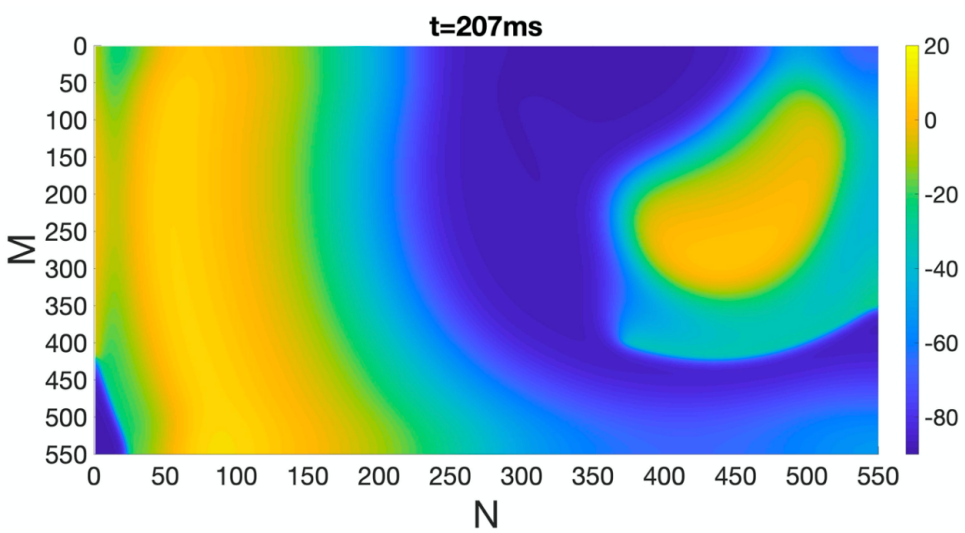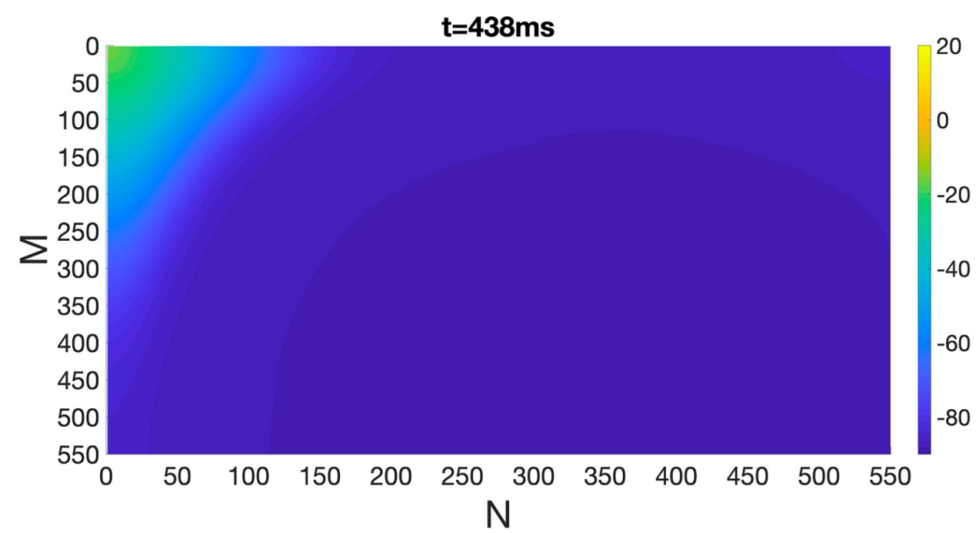

Supplement: S1 Fig — Colorbar indicates Vm (in the units of mV). Snapshots of Vm at time = 0 ms, 20 ms, 207 ms and 438 ms are shown. (PDF) [file pone.0264570.s001.pdf]

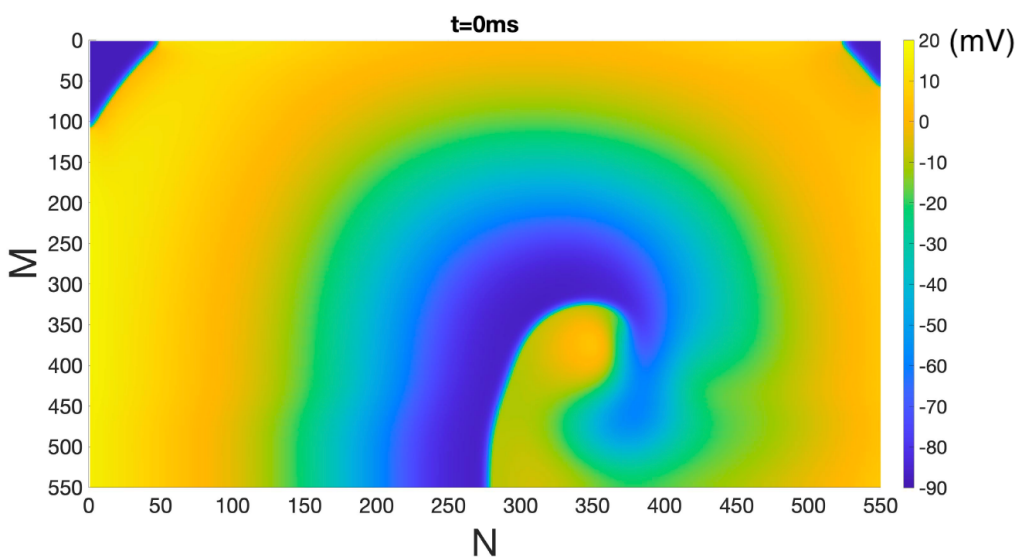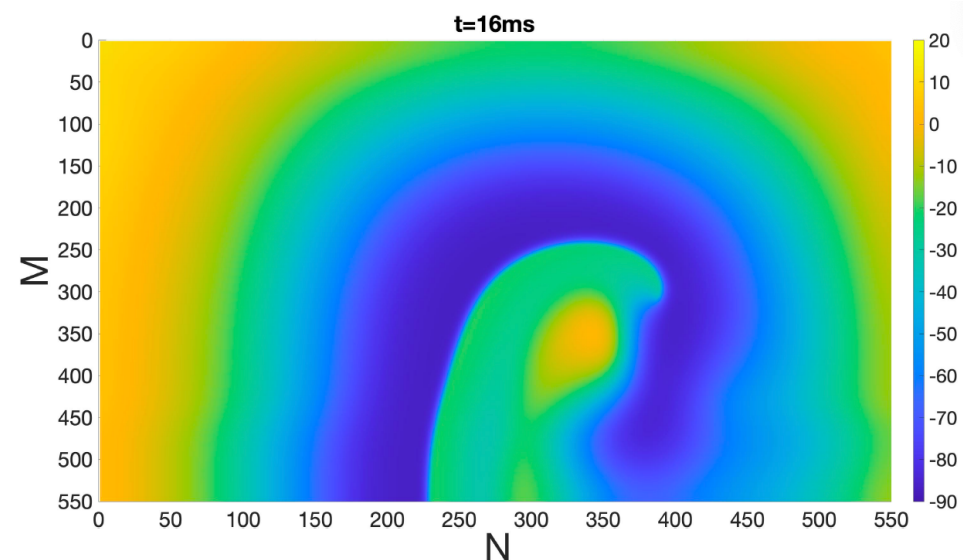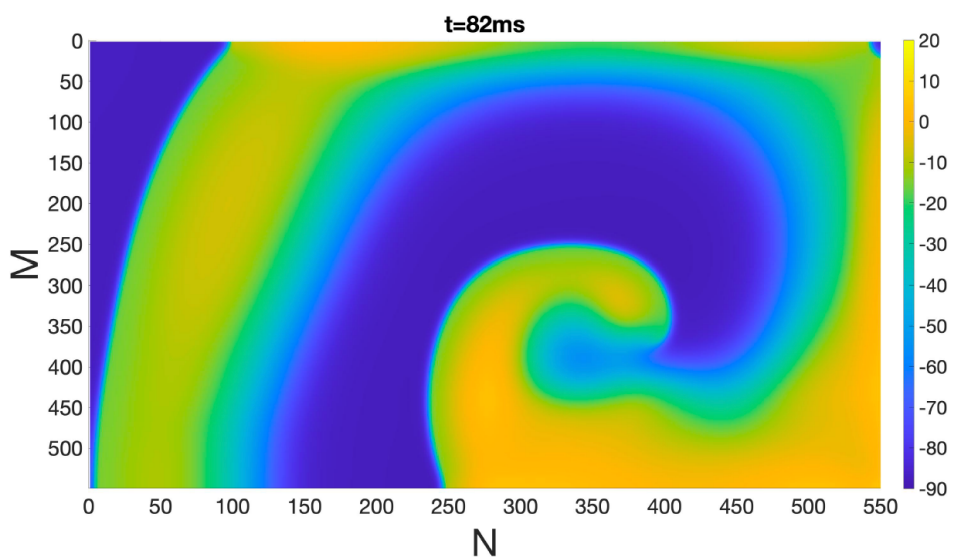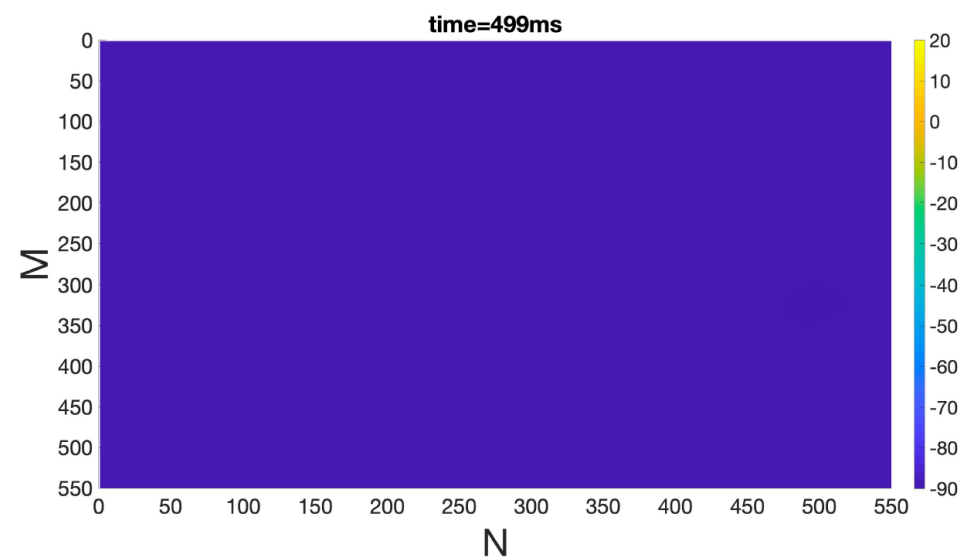

Supplement: S2 Fig — Colorbar indicates Vm (in the units of mV). Snapshots of Vm at time = 0 ms, 16 ms, 82 ms and 499 ms are shown. (PDF) [file pone.0264570.s002.pdf]

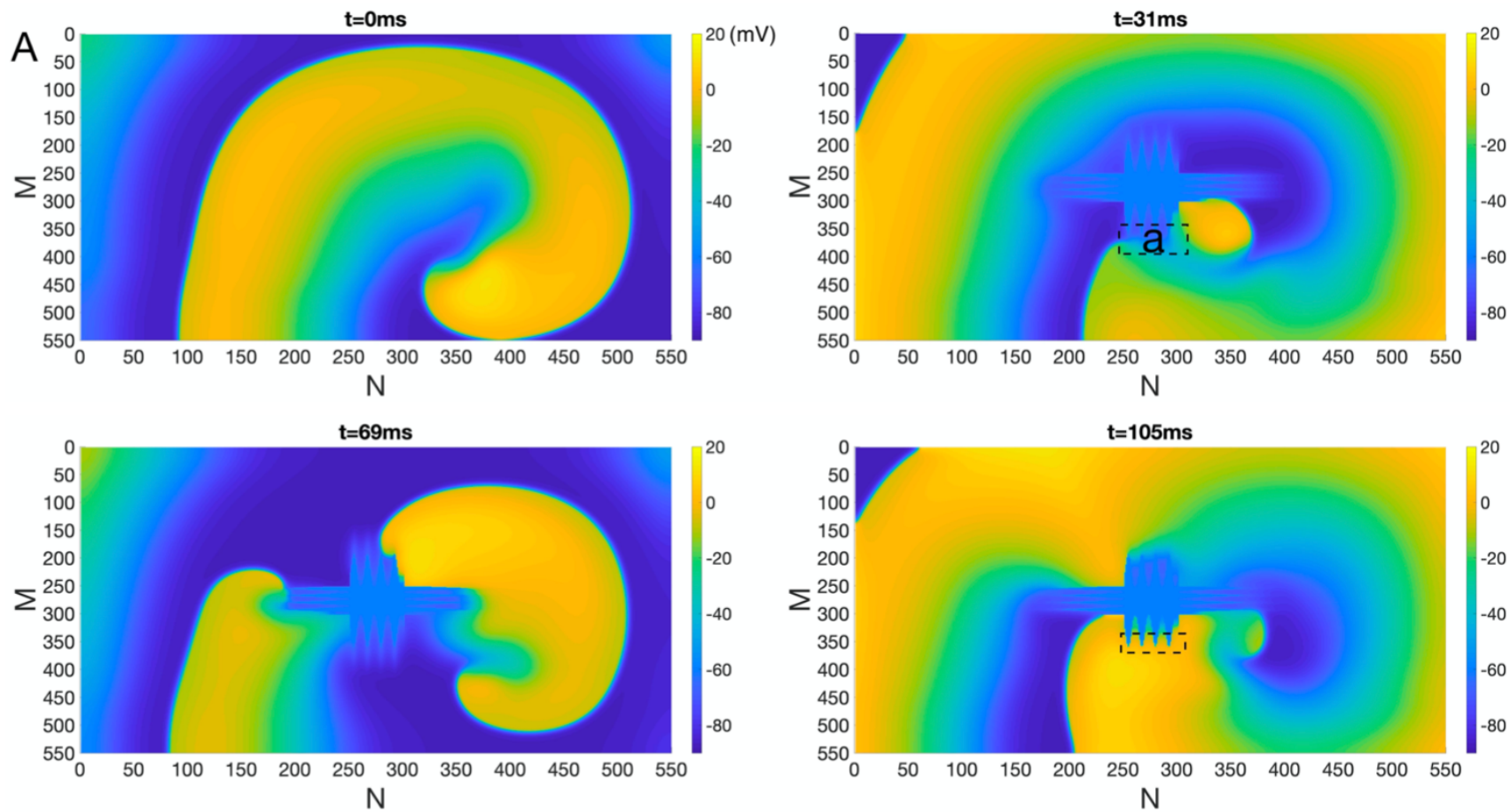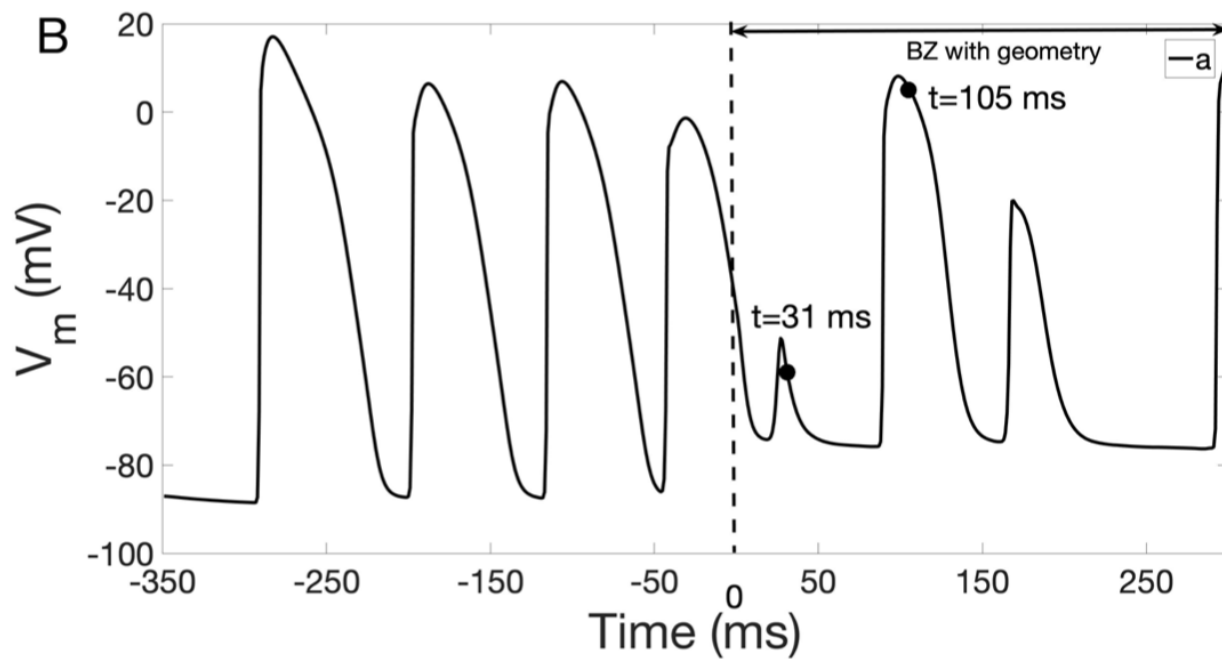

Supplement: S3 Fig — (A) Spiral wave break-up and alternating CB in the presence of BZ with complex geometry in the center. Colorbar indicates Vm (in the unit of mV). Snapshots of Vm at time = 0 ms, 31 ms, 69 ms and 105 ms are shown. Dashed black box indicates the place where wave break occurs. (B) Vm trace of point a. (PDF) [file pone.0264570.s003.pdf]

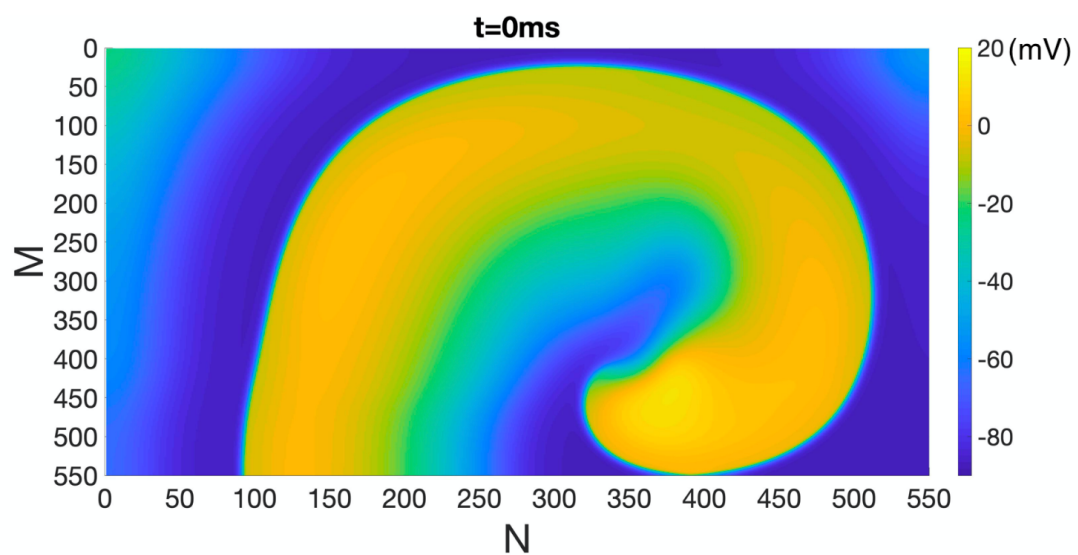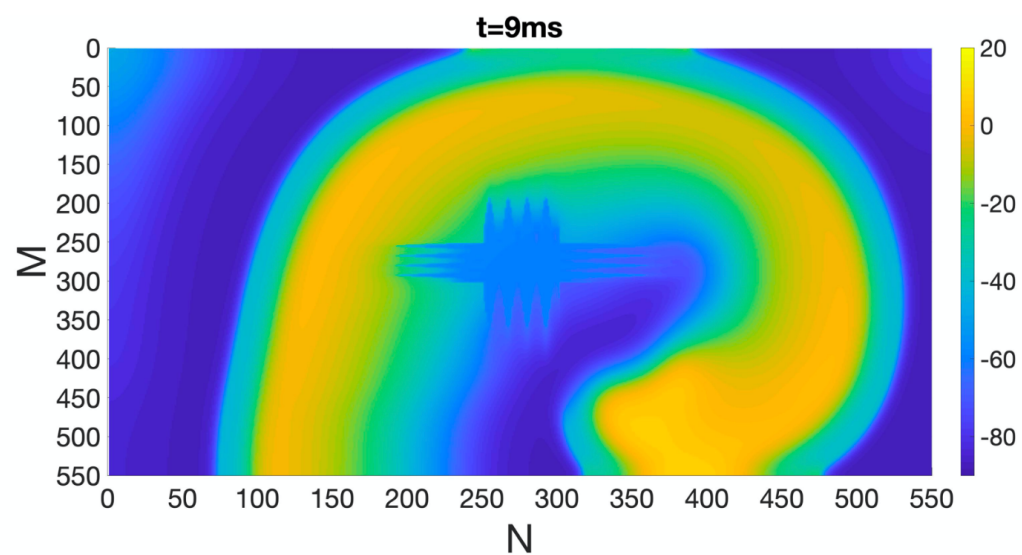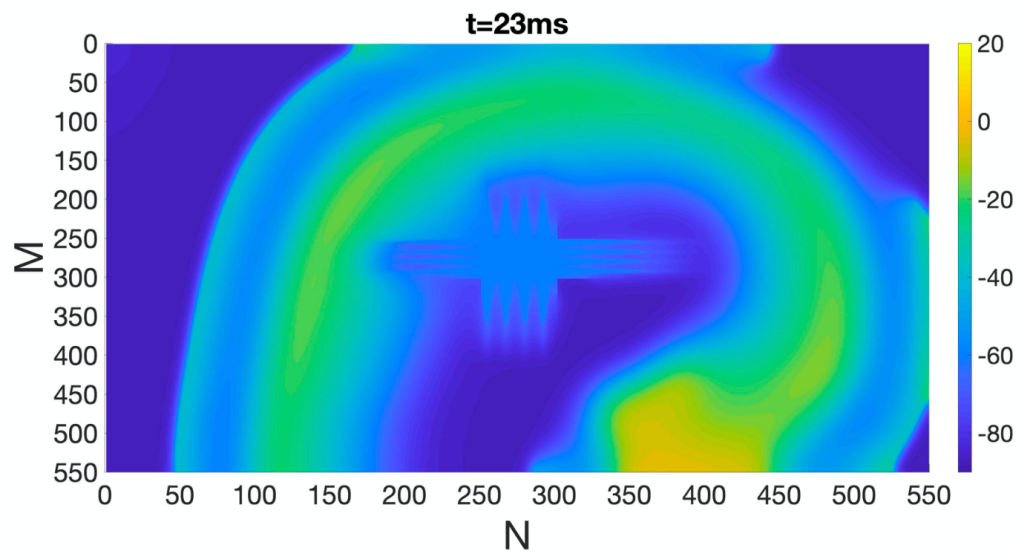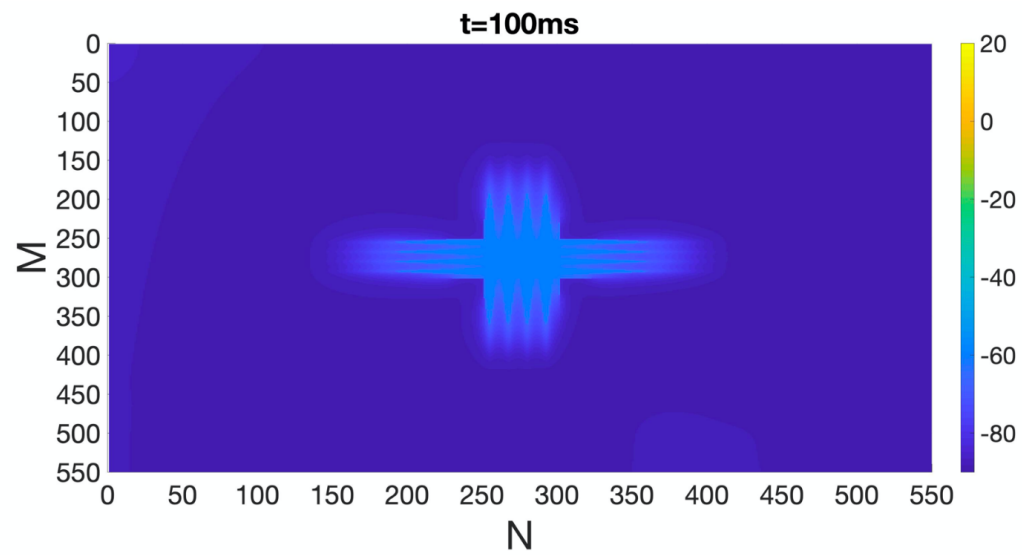

Supplement: S4 Fig — Suppression of reentry in the presence of EpC (dcleft = 8 nm) and BZ with complex geometry in the center of the lattice. Colorbar indicates Vm (in the unit of mV). Snapshots of Vm at time = 0 ms, 9 ms, 23 ms and 100 ms are shown. (PDF) [file pone.0264570.s004.pdf]

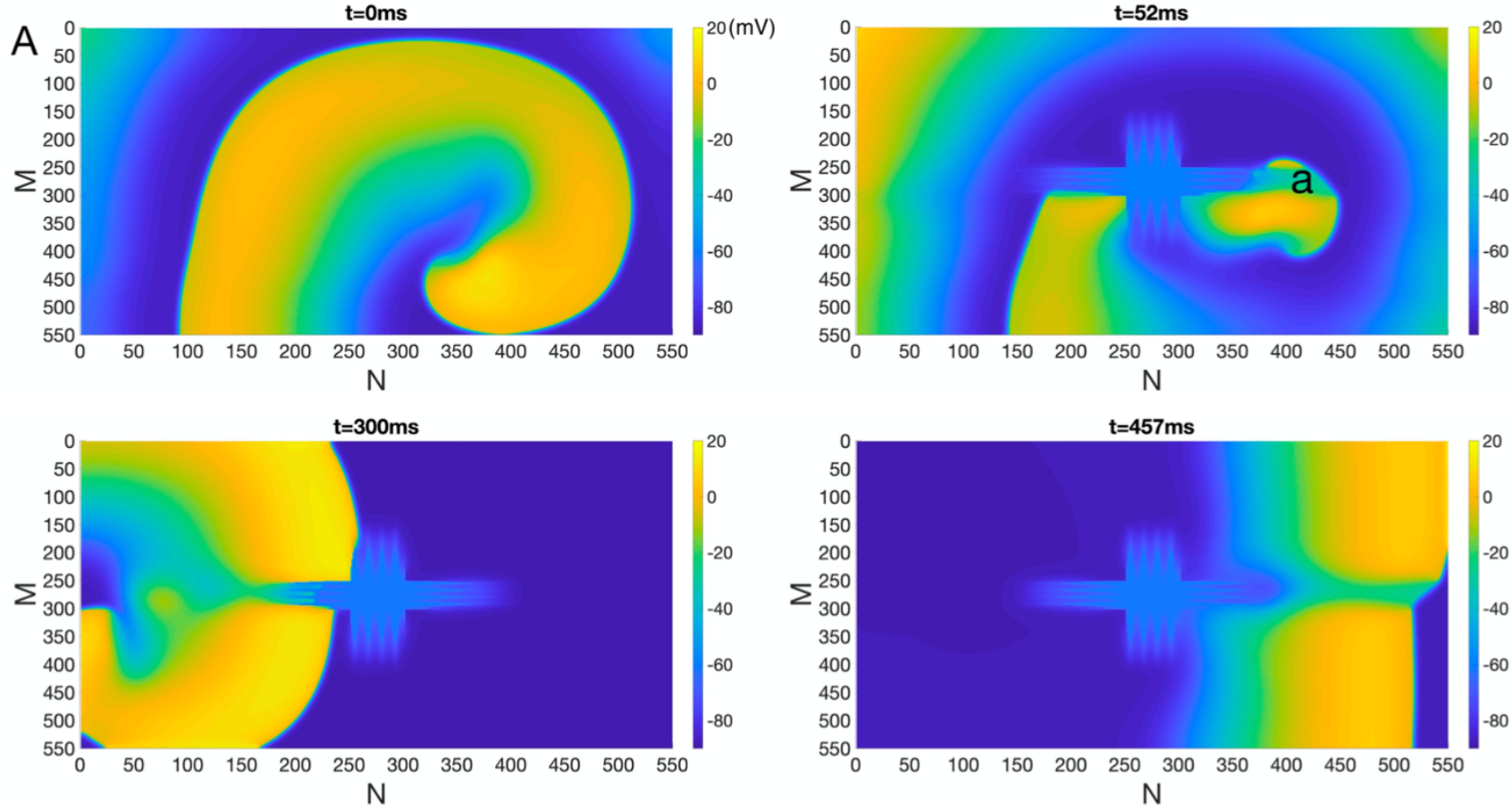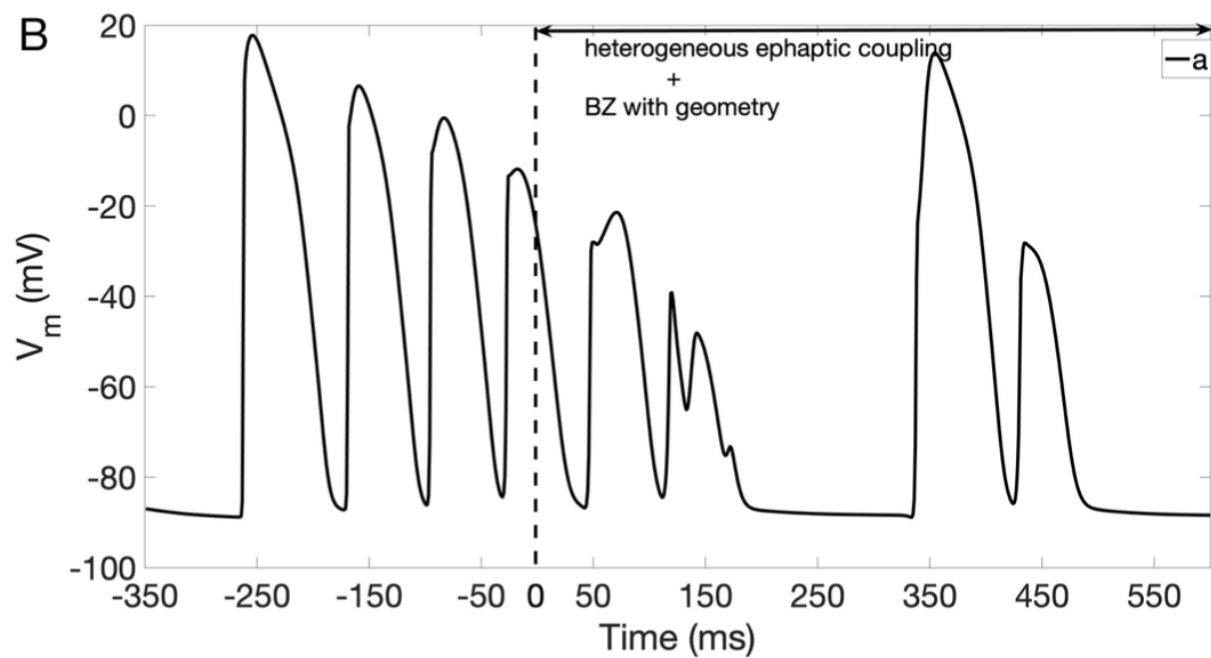

Supplement: S5 Fig — (A) Suppression of reentry in the presence of EpC present in IC BZ and part of NZ and BZ geometry in the center of the lattice. Colorbar indicates Vm (in the unit of mV). Snapshots of Vm at time = 0 ms, 52 ms, 300 ms and 457 ms are shown. (B) Vm trace of point a. (PDF) [file pone.0264570.s005.pdf]
